# Improving plane wave ultrasound imaging through real-time beamformation across multiple arrays

**DOI:** 10.1038/s41598-022-16961-2

**Published:** 2022-08-04

**Authors:** Josquin Foiret, Xiran Cai, Hanna Bendjador, Eun-Yeong Park, Aya Kamaya, Katherine W. Ferrara

**Affiliations:** Stanford University, Palo Alto, CA USA

**Keywords:** Biomedical engineering, Acoustics

## Abstract

Ultrasound imaging is a widely used diagnostic tool but has limitations in the imaging of deep lesions or obese patients where the large depth to aperture size ratio (*f*-number) reduces image quality. Reducing the *f*-number can improve image quality, and in this work, we combined three commercial arrays to create a large imaging aperture of 100 mm and 384 elements. To maintain the frame rate given the large number of elements, plane wave imaging was implemented with all three arrays transmitting a coherent wavefront. On wire targets at a depth of 100 mm, the lateral resolution is significantly improved; the lateral resolution was 1.27 mm with one array (1/3 of the aperture) and 0.37 mm with the full aperture. After creating virtual receiving elements to fill the inter-array gaps, an autoregressive filter reduced the grating lobes originating from the inter-array gaps by − 5.2 dB. On a calibrated commercial phantom, the extended field-of-view and improved spatial resolution were verified. The large aperture facilitates aberration correction using a singular value decomposition-based beamformer. Finally, after approval of the Stanford Institutional Review Board, the three-array configuration was applied in imaging the liver of a volunteer, validating the potential for enhanced resolution.

## Introduction

Ultrasound imaging has been a valuable tool for rapid diagnosis in clinical settings due to its real-time capability, low cost, availability and lack of ionizing radiation. Axial and lateral resolution and contrast are the metrics that are generally defined to gauge image quality. The resolution of an imaging system is defined by its ability to separate two source points. Axial resolution relies on the center frequency and bandwidth of the ultrasound pulse. Increasing the imaging frequency improves the axial resolution but comes at the cost of reduced penetration in the tissue due to frequency-dependent attenuation. At a given imaging frequency, the pulse is minimally affected by propagation in tissue, giving a relatively homogeneous axial resolution throughout the field-of-view. On the other hand, the lateral resolution achieved at a given depth is directly related to the *f*-number (the depth-to-aperture size ratio) which reduces resolving power at larger depths for typical small aperture arrays. If the *f*-number is too large, poor imaging quality is likely to lead to diagnostic failure particularly in the obese patient population^1–4^. Moreover, in the context of abdominal imaging, the presence of heterogeneous tissue layers contributes to acoustic clutter and further reduces image quality^5,6^.

The emergence of high-throughput ultrasound systems allows for full control over a large number of channels (> 256) and offers new opportunities to improve imaging. This technology advance, partly driven by the development of 2D matrix arrays and by ultrafast imaging, gives real-time control over a large number of elements. For 1D-array geometries, it is established that the lateral resolution of an ultrasound system improves when the *f*-number is reduced. This has been demonstrated in several studies through angular or linear sweep of a single array^7^ or by direct imaging with two arrays^8–10^. Even in the presence of heterogeneous tissue layers, a large aperture was shown to improve lesion detectability showing the potential diagnostic benefit of large arrays^7^. Therefore, access to more system channels can be translated into large imaging apertures, improving the image quality while extending the field-of-view.

Early work looking at extending the field-of-view consisted of moving an imaging probe and stitching the recorded images to form a static panoramic view^11^. The wider view of the tissue can be informative for the radiologist but this method does not improve lateral resolution, and motion during acquisition can affect the final composite image. More recently, image compounding with two arrays helped to improve the quality of fetal images^12^. Contrast improvements have been demonstrated by incoherently combining different views of the aorta with one array moving along a fixed circular track above the abdomen^13^. Multi-perspective static aortic imaging was also explored with two curved array transducers^14^. Extending array motion to other dimensions, other groups have also explored the possibility of realizing freehand 3D imaging with a single 1D array^15–17^. However, these approaches preclude real-time capabilities and require image postprocessing to combine incoherently the different views.

Primarily due to technical constraints, previous work looking at increasing the imaging aperture have mainly focused on sweeping an array across the field-of-view and processing the data offline^7,18,19^ or on performing numerical analysis^20^. Although very insightful for the imaging capability of extended aperture geometries in an abdominal imaging scenario, this approach relying on focused beams or full synthetic aperture acquisitions has limited applicability in vivo given the long collection time that would be required.

By combining two commercial arrays placed at fixed locations during data collection, coherent imaging was recently demonstrated^8–10^. For this work, both arrays received simultaneously but transmission was performed one array at a time due to the relatively large gap separating the arrays. In the work from Roberts et al. a synthetic aperture sequence was proposed in order to reduce grating lobes after processing^8^. To maintain a high frame rate, Peralta et al. implemented a plane wave imaging sequence^9,10^.

Combining individual arrays inevitably creates a partially sampled aperture with physical gaps. The lack of spatial information within these gaps results in grating lobes that can be detrimental to the image quality^8–10^. With a large array, this situation could also be encountered in vivo if bone or air blocks a subset of the aperture. The problem of blocked elements in ultrasound imaging is not new and several methods have been proposed to recover the missing information or to reduce grating lobes. An adaptive beamforming method using information from multiple receive beams and the total least-squares method was proposed to estimate and reduce unwanted sidelobe contributions in the presence of blocked elements^21,22^. From full synthetic aperture datasets, adaptive weighing of nonblocked elements channel data was proposed to rescale attenuated spatial frequencies and reduce side lobe levels^23^. In the case of a flat linear aperture with missing elements, grating lobe reduction is possible after missing channel data is estimated from neighboring elements^24^. In the case of relatively large gaps (several elements), it is also possible to make use of redundancy of spatial information between pairs of transmit and receive elements^8^, although full compensation is possible when the gaps are smaller than the active sub-apertures. However, optimal compensation is achieved by acquiring complete synthetic aperture datasets to cover all the available pairs of transmit-receive elements, requiring long acquisition time and eliminating ultrafast applications.

The design of arrays with unusual geometries is a slow and costly process for which simulation is required to predict the imaging performances. Repurposing commercial arrays to mimic a very large aperture is an interesting opportunity to quickly investigate in situ the possibilities of such geometries. 3D printing has expanded tremendously in recent years due to the low cost and availability of consumer grade printers and its use to create customized manifold for arrays is particularly attractive. Contrary to multi-transducer approaches described previously, we chose to simplify the design and fix the geometry after assembly. A 3D printed manifold was designed to allow stacking of multiple arrays with a fixed location with respect to one another. After assembly, calibration is required to calculate the array positions with sub-wavelength precision but after the positions are retrieved, the assembly can be utilized as a standard array. Depending on the application, the manifold can be modified to change the overall geometry and arrays can be added or omitted thanks to the stackable feature. This approach has the advantage of being practical in a clinical setting where the assembly can directly image without the need for a mechanical scan or a recalibration of the positions if the arrays are able to move independently from one another.

Large apertures comprising several hundred elements require careful consideration regarding the imaging sequence: a typical focused beam sequence will see its acquisition time increase linearly with the number of elements and reach a point where tissue motion will degrade image quality. Plane wave imaging, often termed coherent plane wave compounding, allows ultrafast image collection reaching kHz rates and is capable of imaging performance similar to conventional focused beams^25–27^. Ultrafast acquisitions are thus more suited to the large field-of-view accessible with large arrays. Furthermore, many novel active areas of research such as transient elastography^28,29^, assessment of blood flow^30–32^, functional imaging of the brain^33–35^ or ultrasound localization microscopy^36–39^ are a direct extension of ultrafast imaging and could directly benefit from larger imaging apertures.

In this work, we are employing plane wave (PW) imaging to ensure fast acquisition as previously reported for a multi-transducer assembly^9,10^. Generating plane waves with multiple transducers with a coherent wavefront presents several advantages: the assembly can be used for ultrafast imaging (plane or diverging waves) mimicking an extended aperture with gaps. Although image resolution is improved straightforwardly by reducing the *f*-number (e.g. increasing the imaging aperture at a given depth), the full benefit of the improvement is still hampered by the presence of aberrating layers and/or array blockage. By simplifying the acquisition sequence to reproduce a single aperture PW sequence, the ability to apply fast aberration correction methods such as the Singular Value Decomposition (SVD) beamformer^40^ is possible.

In this work, we explored such a configuration by combining three commercial phased arrays and tested the improvements achieved by a large aperture with 384 elements and a 10 cm lateral aperture imaging in real-time. We investigated the setup in vitro on wire targets and on a calibrated commercial phantom and in vivo imaging the liver. To compensate for these gaps, we adapted an auto-regressive (AR) filter in order to predict the receive signals on virtual elements extending the physical arrays and filling the gaps. The AR filter was originally applied by Shin et al. for ultrasound clutter reduction and to account for missing channels^41^. We also investigated the effect of an aberrating layer with the three-array assembly and the improvements achieved after applying the SVD beamformer.

## Results

### Lateral resolution and field of view

**Figure 1:**
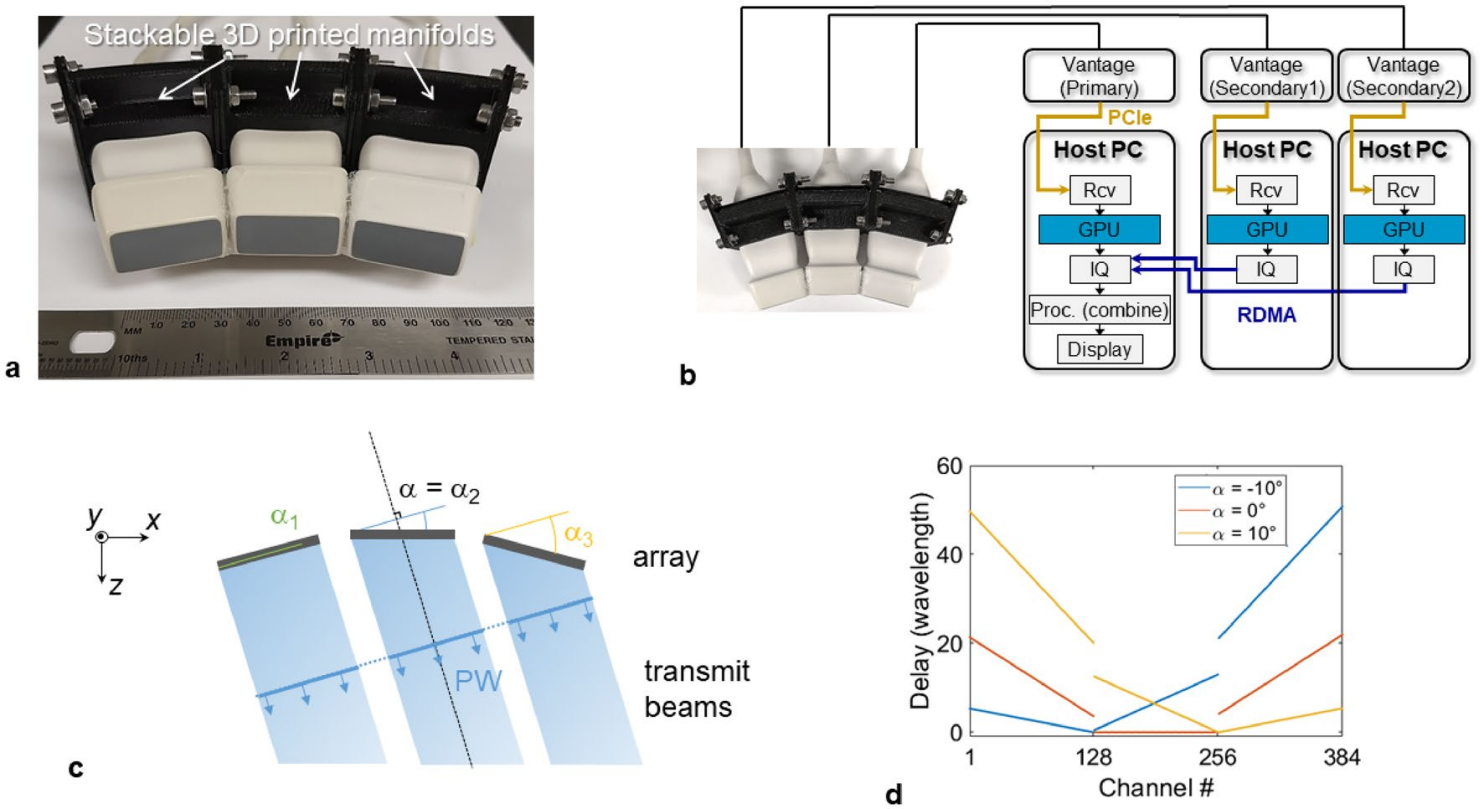
Imaging with multiple arrays. (**a**) Picture of the three-array assembly with its modular 3D-printed stackable manifold. The slight concave geometry minimizes gaps between the arrays. (**b**) Experimental workflow: each array is connected to a separate Vantage system (part of the 1024 channel volumetric package) where partial GPU beamforming allows efficient processing and real-time display. All the ultrasound systems share the same clock enabling synchronous transmit and receive operation. (**c**) All arrays are used to transmit plane waves and all arrays are used on receive ultrasound signals. (**d**) Delay signals sent to generate plane waves taking into account the angle of each array with respect to the central array.

**Figure 2:**
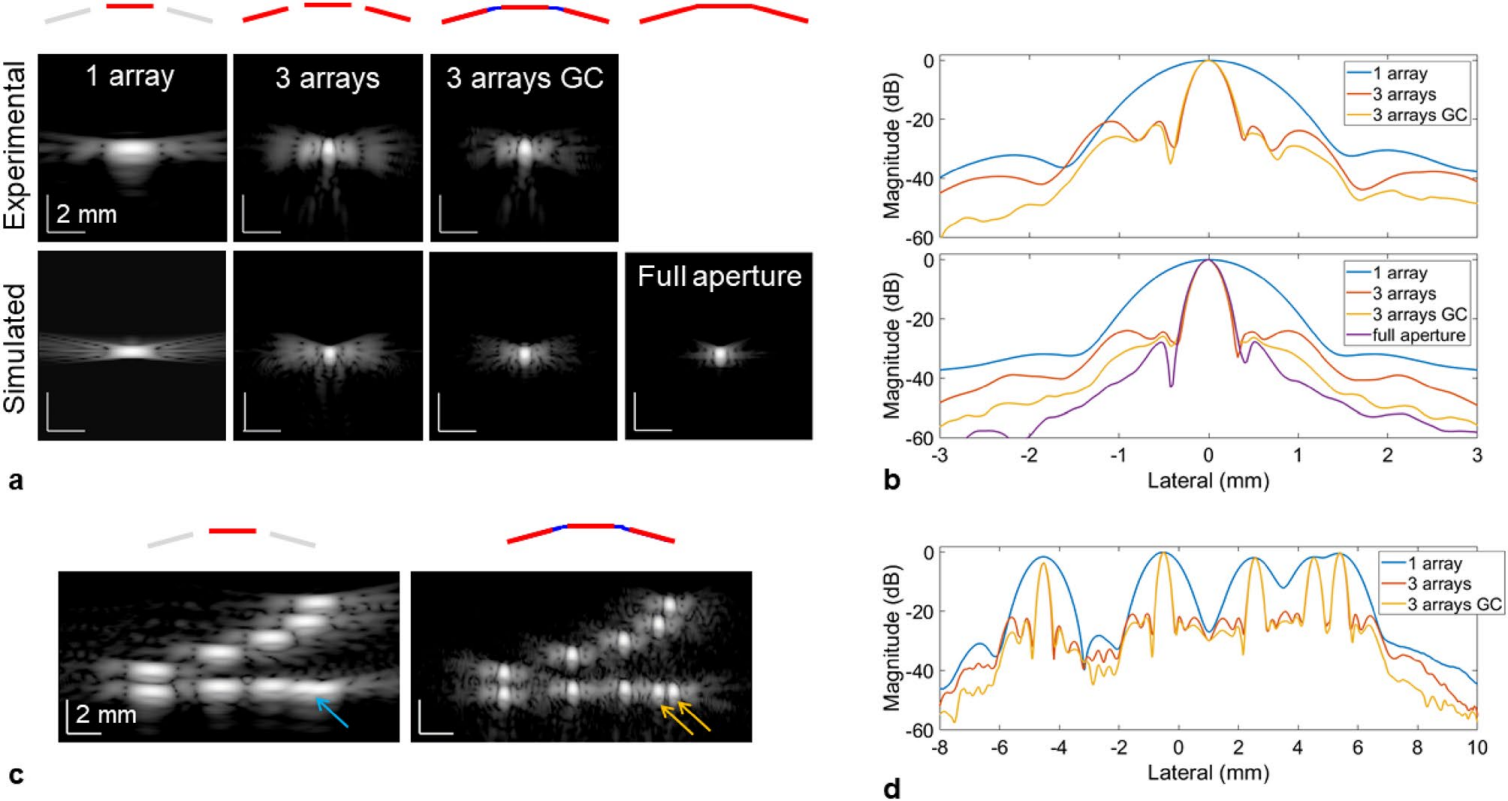
Expanding the imaging aperture effectively improves the resolution as demonstrated by evaluating the point spread function (PSF). (**a**) Comparison of experimental (top row) and simulated (bottom row) PSF for a 25 µm wire located at a depth of 100 mm. From left to right, the columns correspond to the PSF reconstructed using the central array only, all three arrays, all three arrays with gap compensation and an equivalent fully populated aperture (i.e. no gaps). (**b**) Lateral cross-section of the PSF displayed in (**a**). (**c**) Image of wire targets with known spacing (lateral spacing 1–4 mm with 1 mm increment, axial spacing 1–5 mm with 1 mm increment) located at a depth of 100 mm using the central array only (left) and all three arrays with gap compensation (right). The wires spaced 1 mm laterally cannot be resolved laterally with one array (blue arrow) but are fully separated using three arrays (orange arrows). (**d**) Lateral cross-section of the targets aligned horizontally displayed in (**c**). For (**a**) and (**c**) the dynamic range is 60 dB.

First, we evaluated the lateral resolution improvement with the three-array assembly depicted in Fig. 1a. The arrays (P6-3, ATL) were arranged to minimize the gaps and formed a piece-wise concave aperture (15° between adjacent arrays) with a lateral extent of 99.4 mm. To optimize the data collection and processing workflow, each individual array was connected to a programmable ultrasound system (Vantage 256, Verasonics, Kirkland, WA) synchronized as described in Fig. 1b. In this configuration, each system host was performing a partial reconstruction with a Graphical Processing Unit (GPU; Titan RTX, Nvidia, Santa Clara, CA) for 128 channels and sending the beamformed data to the primary system for real-time display. This setup facilitated reconstructing an image of 120 × 130 mm (600 × 650 pixels) for a single plane wave transmit in 1 ms. The imaging sequence consisted of transmitting *N* PW utilizing the entire aperture (Fig. 1c,d). To compare the differences as compared with imaging with a single array, a full synthetic aperture acquisition was collected with the central array only. The evaluation of the experimental point-spread-function (PSF) on thin wire targets (25 µm), which is shown here at a depth of 100 mm (Fig. 2a), demonstrated a significant reduction of the main lobe lateral size after extending the imaging aperture. From the lateral cross section of the PSF (Fig. 2b), the lateral resolution (defined as the full-width half-maximum) was measured at 0.37 mm compared with 1.27 mm using a single array. These results were in agreement with simulations performed with the same array geometry where the lateral resolution was 0.34 mm and 1.18 mm, respectively. Note that a full synthetic aperture sequence, yielding the best lateral resolution, was employed for the 1-array results. The equivalent *f*-number (ratio of depth over lateral aperture) is reduced by a factor of 3.49 using the three arrays. It is also interesting to note that at this depth, the lateral resolution becomes similar to the axial resolution, measured at 0.4 mm (Supplementary Fig. 1), which only relies on the transmitted waveform and transducer material characteristics.

### Gap filling algorithm and performance

Grating lobes are expected due to the inter-array gaps and were visible here. To reduce this effect, an auto-regressive filter was employed to estimate signals on virtual elements positioned to fill the inter-array gaps. The method yielded a reduction of the gap-related grating lobes with an average difference of − 5.2 dB (from − 22.2 to − 27.4 dB) measured experimentally (Fig. 2b). On simulated data, the reduction was measured at − 6.6 dB (from − 23.9 to − 30.5 dB). The PSF obtained with a continuous aperture (i.e. as if the virtual elements were part of the array and actively receiving signals) showed a similar main lobe profile and further reduced grating lobe levels down to − 39.2 dB.

The improvement in lateral resolution is also demonstrated on wire targets with a set spacing both in the axial and lateral directions (Fig. 2c; 1–4 mm laterally with 1 mm increments; 1–5 mm axially with 1 mm increments). At a depth of 100 mm, the closest wires laterally (1 mm gap) cannot be resolved using a single array but are fully separated when using the three arrays (Fig. 2d).

**Figure 3:**
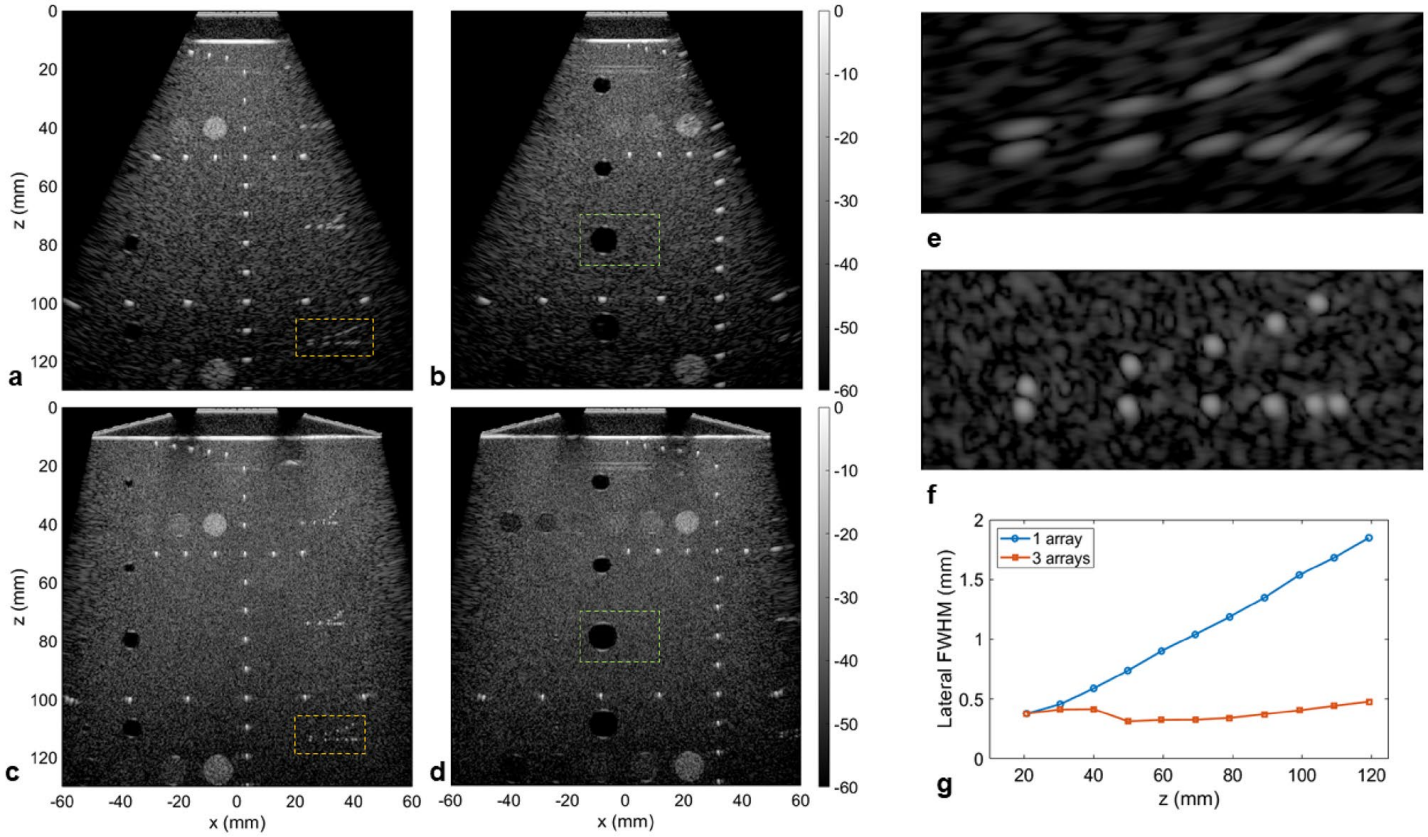
Extended aperture imaging on a calibrated commercial phantom (CIRS 054GS). (**a**–**d**). Images of the phantom utilizing only the central array ((**a**) above the wire targets; (**b**) above the hypoechoic cysts) or all three arrays ((**c**) above the wire targets; (**d**) above the hypoechoic cysts) for 91 plane waves (− 30° to 30°). (**e**, **f**) The expanded view of the deeper set of wire targets (orange rectangle in (**a**) and (**c**), located at a depth of 110 mm) shows the significant improvement in term of lateral resolution going from one array (**e**) to 3 arrays (**f**). The dynamic range is 60 dB for all images. Note that an agar/glycerol phantom standoff (speed of sound 1540 m/s) is inserted between the array assembly and the top surface of the phantom. (**g**) Comparison of the lateral resolution (full-width at half maximum) as a function of depth for the set of wires located at the center of (**a**) and (**c**).

Measurements on a commercial phantom (model 054GS, CIRS) showed a similar improvement going from imaging with only the central array (Fig. 3a,b) to the array assembly (Fig. 3c,d). Here, the enhanced field-of-view combined with significantly improved lateral resolution across the image revealed an overall clearer view of the various targets. The improvement was particularly noticeable at larger depths, as seen on the off-centered wire targets (Fig. 3e,f) which were completely resolved with the extended aperture. By measuring the lateral resolution from wires located at depths ranging from 20 to 120 mm (Fig. 3g), the effect of the *f*-number was clearly apparent for the single array case with a lateral resolution linearly degrading with depth (i.e. doubling every 28 mm). With the three-array assembly resulting in an effective lateral aperture of 99.4 mm, the lateral resolution was improved for deep targets with a slower degradation rate (i.e. doubling every 99.4 mm).

Finally, on the bright wire targets, the gap compensation method pushed the grating lobes to a level below the speckle background and were only visible for wires on the edge of the image where compensation was difficult. It is also interesting to note the unusual elongated shape of the wires associated with an axial resolution that was inferior to its lateral counterpart, which is rarely seen in ultrasound imaging.

### Contrast and contrast to noise ratio

**Figure 4:**
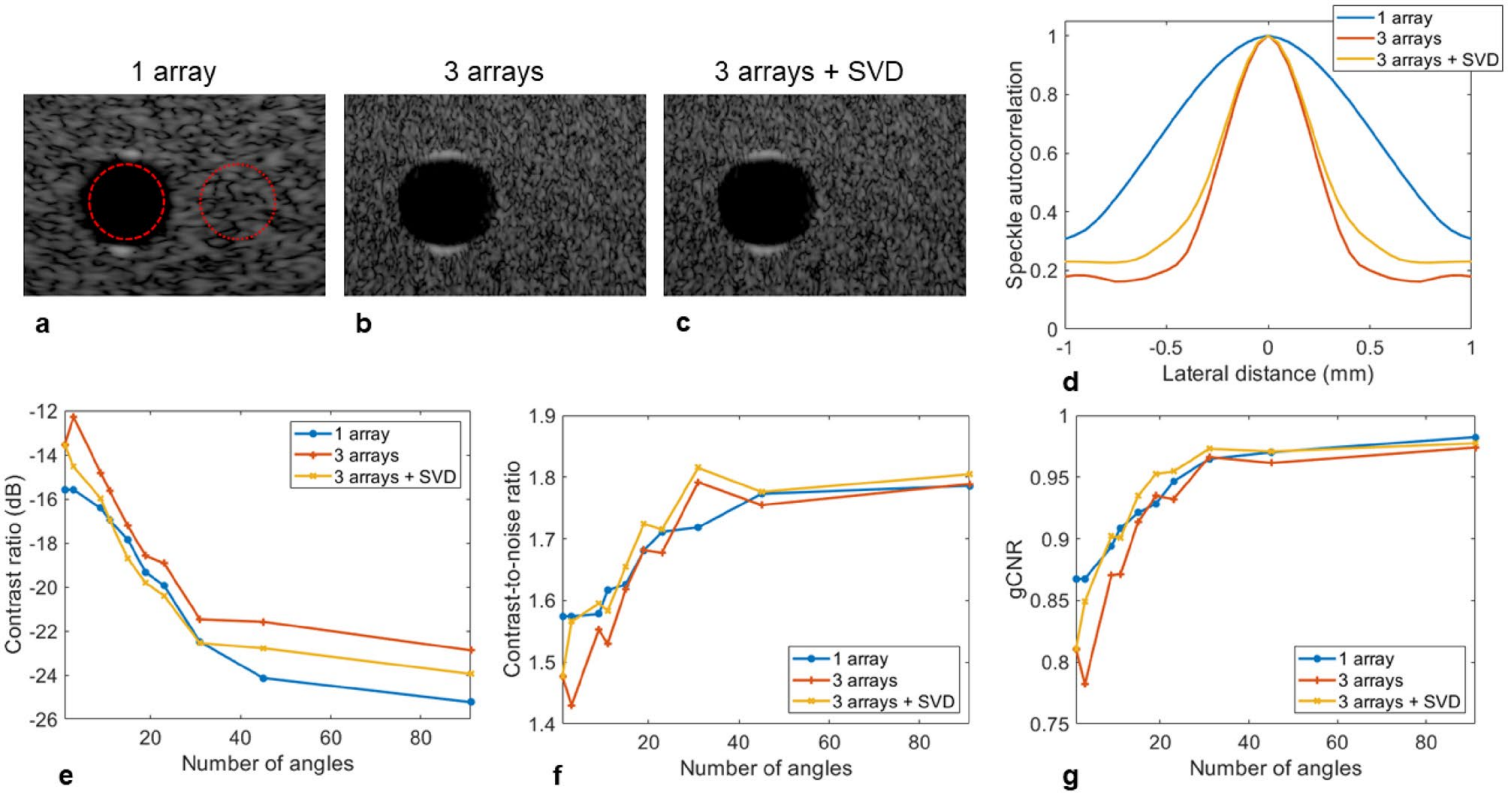
Quantification of contrast on the hypoechoic cyst located at 80 mm as a function of the number of plane waves. (**a**–**c**). Expanded view of the hypoechoic region located at 80 mm (green rectangle in Fig. 3 (**b**) and (**d**)) going from one array (**a**) to three arrays (**b**) and after applying the SVD beamformer (**c**). The ROIs indicate the inside and outside areas selected to calculate the contrast metrics. The dynamic range is 60 dB for all images. (**d**) The autocorrelation of the speckle as a function of the lateral distance confirms the change in speckle aspect utilizing three arrays with reduced lateral correlation distance. (**e**) The contrast ratio as a function of the number of angles shows a slight degradation going from one to three arrays that is partially compensated by the SVD beamformer. (**f**, **g**) The contrast-to-noise ratio (**f**) and generalized contrast-to-noise ratio (**g**) as a function of the number of angles are very similar between one and three arrays, especially for a higher number of angles. A small degradation is seen for three arrays for a small number of angles but is compensated when applying the SVD beamformer.

We focused the analysis of the contrast metrics as a function of the number of plane waves on the cyst-like structure located at a depth of 80 mm in Fig. 3b,d. An expanded view of the cyst is given in Fig. 4a–c along with the regions delimiting the pixels used in the calculations. Besides the clear change in the aspect of the surrounding speckle, the boundaries of the cyst were better defined with the extended aperture. Upon analysis of the autocorrelation of the background speckle, the lateral correlation distance for the three-array assembly (Fig. 4d) decreased from 1.38 to 0.55 mm (for an autocorrelation magnitude of 0.5). As a result, the background speckle appears finer in comparison to the single array.

The contrast ratio (Fig. 4e) appeared to slightly degrade when using three arrays with a contrast reaching − 22.9 dB compared to − 25.2 dB for the central array only (91 PW). The contrast-to-noise ratio (Fig. 4f) was again slightly degraded for a small number of PWs but similar with a higher number of PWs reaching 1.79. A comparable behavior was observed for the generalized contrast-to-noise ratio (Fig. 4g). We then applied the SVD beamformer to further check the results obtained for the three-array case. Minimal improvements were seen as a result, confirming the proper image formation. The small correction applied by the SVD beamformer could be linked to a small speed-of-sound mismatch between the agar/glycerol standoff and the phantom.

For comparison, the three contrast metrics are also provided for cyst-like structures located at depths of 55 and 110 mm for 91 PW (Table 1). At 55 mm, the observations are similar to those at a depth of 80 mm with slightly degraded contrast. However, at 110 mm the three-array assembly outperforms the single array case, even without the application of the SVD beamformer, with an improvement of 3.4 dB for the contrast, 0.34 for the CNR and 0.19 for the gCNR.

**Table 1:** Evaluation of contrast ratio (CR), contrast-to-noise ratio (CNR) and generalized contrast-to-noise ratio (gCNR) after imaging with the central array only or with all three arrays on the commercial phantom.

	1 array	3 arrays	3 arrays + SVD
**Cyst 55 mm**			
Contrast (dB)	− 25.5	− 22.7	− 24.2
CNR	1.80	1.73	1.78
gCNR	0.96	0.96	0.96
**Cyst 80 mm**			
Contrast (dB)	− 25.2	− 22.9	− 24.0
CNR	1.79	1.79	1.81
gCNR	0.98	0.97	0.97
**Cyst 110 mm**			
Contrast (dB)	− 12.2	− 15.6	− 16.1
CNR	1.27	1.65	1.69
gCNR	0.69	0.88	0.88

Quantification is done on cyst-like structures at depths of 55, 80 and 110 mm for 91 PWs (− 30° to 30°).

### Aberration correction

**Figure 5:**
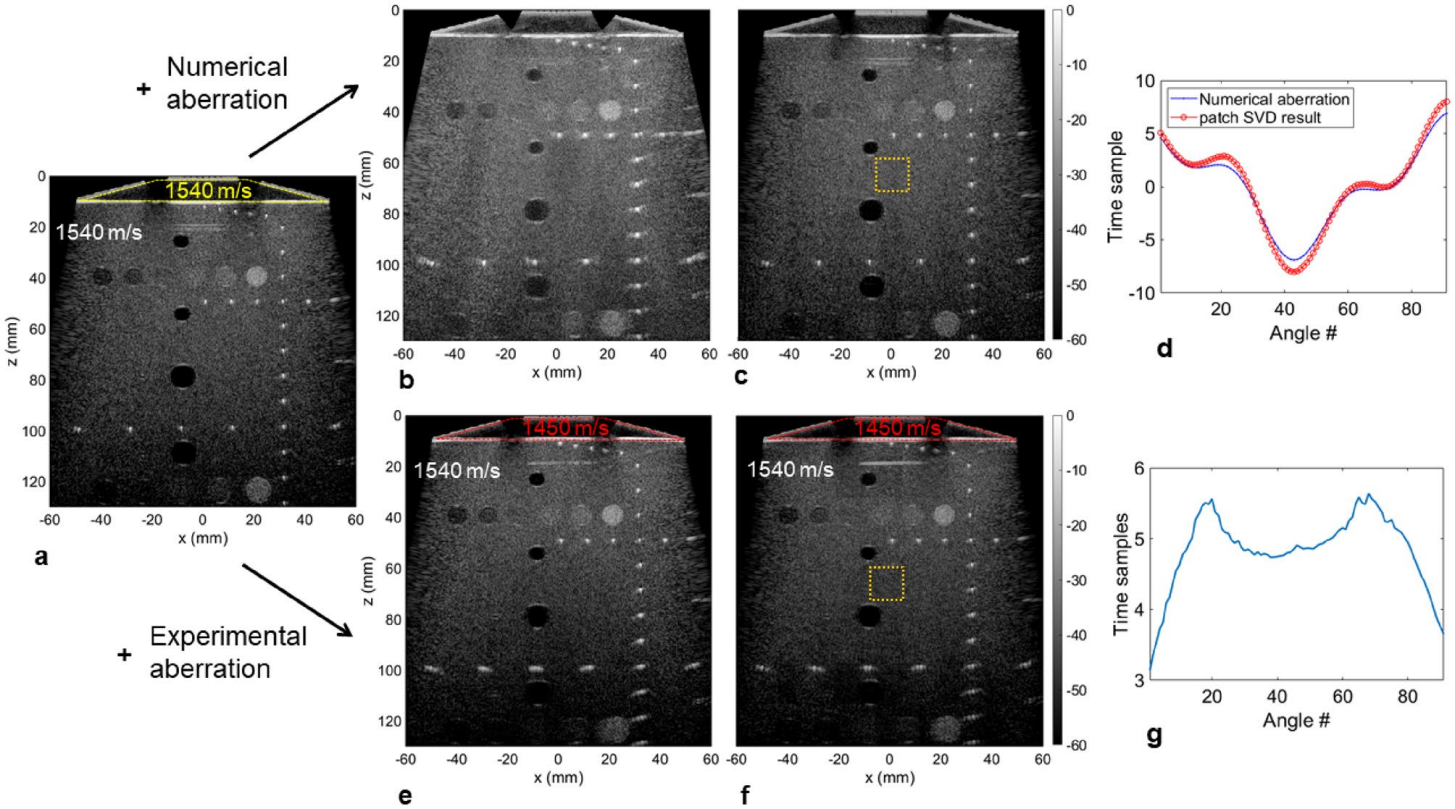
Evaluation of aberration correction on the array assembly with the SVD beamformer on the calibrated commercial phantom (CIRS 054GS). (**a**) Delay-and-sum (DAS) image of the phantom with proper speed of sound matching between the arrays and the top flat surface of the phantom (the yellow trapezoidal contour highlights the agar/glycerol standoff). (**b**) A numerical aberration is applied on the receive data resulting in image degradation. (**c**) The application of the SVD beamformer retrieves the original image. (**d**) Comparison of the aberration estimated from the SVD processing (red) to the numerical aberration applied to the receive data (blue) as a function of the angle number. The isoplanatic patch used for the estimation is indicated by the orange square in (**c**). (**e**) The standoff is replaced with a water/ethanol solution (35/65 volume ratio) reducing the speed of sound to 1450 m/s and degrading the DAS image. (**f**) Image degradation is reduced after applying the SVD beamformer. (**g**) Aberration estimated from the SVD processing as a function of the angle number. The isoplanatic patch used for the estimation is indicated by the square in (**f**). The dynamic range is 60 dB for all images.

We further investigated the effect of aberrations on the array assembly on the commercial phantom by introducing numerical delays to the receive signals (Fig. 5a,b). This one-way phase screen (only applied on receive) degraded the overall image quality (Fig. 5b) with reduced contrast and a broadening of the previously well localized wire targets. After applying the SVD beamformer, the image was completely recovered (Fig. 5c) with an aspect similar to the non-aberrated image. From a given isoplanatic patch, the estimated phase screen was retrieved (Fig. 5d) and correlated well with the applied aberration law. Finally, we replaced the agar/glycerol standoff (1540 m/s) between the arrays and the phantom by a water/ethanol solution (35/65 volume ratio; 1450 m/s) effectively introducing an aberrating layer with a speed of sound similar to fat. As seen previously, it resulted in a degraded image that was here more apparent at larger depths (Fig. 5e). Improvements were noticeable after applying the SVD beamformer (Fig. 5f). Moreover, the retrieved aberration law for a patch in the center of the image (Fig. 5g) revealed a trapezoidal shape that intuitively corresponds to the shape of the aberrating layer.

**Figure 6:**
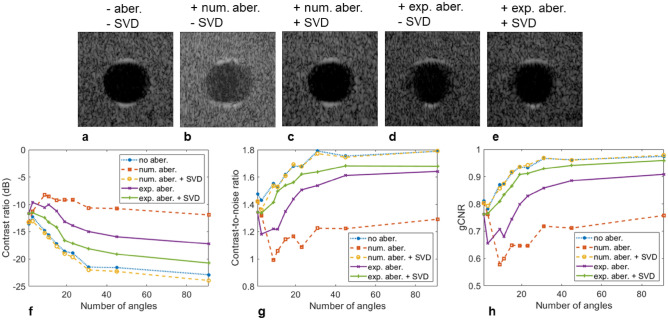
Quantification of contrast on the hypoechoic cyst located at 80 mm (see Fig. 5) as a function of the number of plane waves when aberrations are introduced. (**a**–**e**) Expanded view of the cyst without aberration (**a**), after introducing a numerical aberration before (**b**) and after (**c**) applying the SVD beamformer, after introducing an experimental aberrating layer before (**d**) and after (**e**) applying the SVD beamformer. The dynamic range is 60 dB for all images. (**f**–**h**). The contrast ratio (**f**), contrast-to-noise ratio (**g**) and generalized contrast-to-noise ratio (**h**) as a function of the number of angles shows a clear degradation in the presence of aberration.

A more detailed analysis of the correction effect on the contrast is given in Fig. 6. Close-up views of the cyst located at a depth of 80 mm (Fig. 6a–e) showed a better view of the improvements generated by the SVD beamformer. When introducing the random numerical aberration (Fig. 6b), the overall contrast and the cyst boundaries were affected but the correction applied by the SVD beamformer recovered the initial image (Fig. 6c). The experimental aberrator (Fig. 6d) blurred the cyst lateral boundaries but this was corrected by the SVD beamformer (Fig. 6e). Quantifying the contrast metrics as a function of the number of angles (Fig. 6f–h) demonstrates the strong effect of the aberration reducing the CR by 5.7 dB experimentally (Table 2) (11 dB with the applied numerical aberrating screen), the CNR by 0.15 (0.5 numerically) and the gCNR by 0.07 (0.21 numerically). On the numerical phase screen, the SVD beamformer performed well (regardless of the number of angles), recovered the non-aberrated image and slightly improved the CR (for similar reasons as discussed for Fig. 4). On the experimental data, the SVD beamformer correction improved the CR by 3.5 dB, the CNR by 0.04 and the gCNR by 0.05. Interestingly, in the presence of an experimental aberration, all three contrast metrics were degraded going from a single PW to a small number of PWs with the worst case being three PWs. This results from the fact that with only three transmissions, a different array will illuminate the cyst for each PW, maximizing the effect of the aberration. With more PWs this effect is gradually reduced by averaging but at least 15 PWs are necessary to match the contrast obtained with a single PW (in the case of the experimental aberration). The SVD beamformer breaks this dynamic and the metrics improved with the number of PWs. This effect was even more pronounced for the numerical aberrator where 91 PWs reduced the CR compared to a single PW. Improvements are also measured for the cyst-like structures located at 55 and 110 mm (Table 2).

**Table 2:** Evaluation of contrast ratio (CR), contrast-to-noise ratio (CNR) and generalized contrast-to-noise ratio (gCNR) in the presence of aberrations on the commercial phantom.

	w/o aberration	w/ aberration	w/ aberration + SVD
**Cyst 55 mm**			
CR (dB)	− 22.7	− 20.8	− 23.9
CNR	1.73	1.75	1.80
gCNR	0.96	0.94	0.97
**Cyst 80 mm**			
CR (dB)	− 22.9	− 17.2	− 20.7
CNR	1.79	1.64	1.68
gCNR	0.97	0.91	0.96
**Cyst 110 mm**			
CR (dB)	− 15.6	− 9.4	− 13.7
CNR	1.65	1.19	1.46
gCNR	0.88	0.65	0.79

Quantification is done on cyst-like structures at depths of 55, 80 and 110 mm for 91 PWs (− 30° to 30°). Aberrations are introduced by a layer of water/ethanol solution with a speed of sound of 1450 m/s between the arrays and the CIRS phantom (1540 m/s).

### In vivo imaging performance

**Figure 7:**
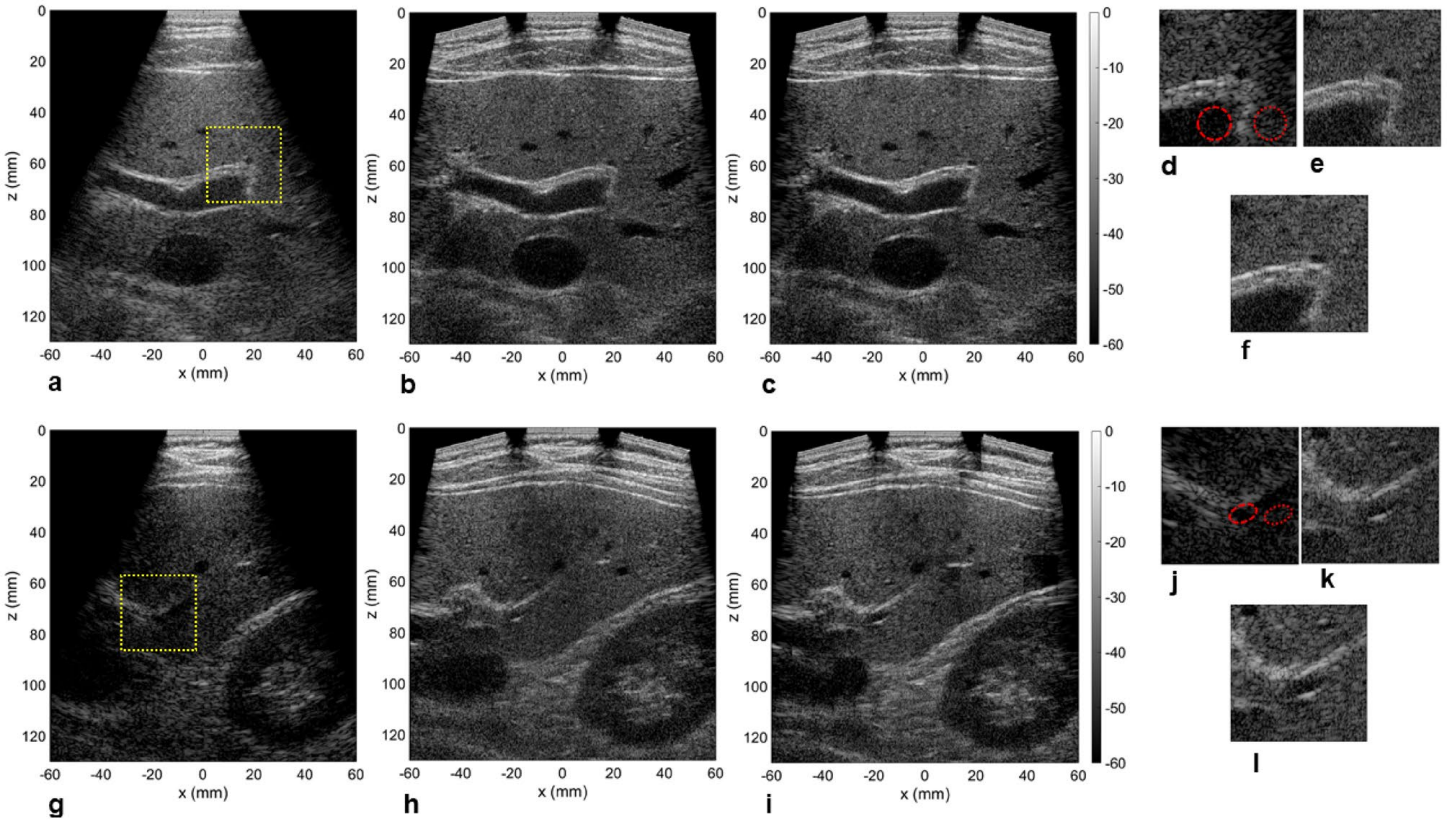
Imaging of liver on a volunteer using the central array (**a**, **g**), all three arrays (**b**, **h**) and after applying the SVD beamformer (**c**, **i**). The close up view indicated in (**a**) and (**g**) by a yellow square is displayed for all three cases in (**d**) and (**j**) (one array), (**e**) and (**k**) (three arrays) and (**f**) and (**l**) (three arrays + SVD). The dynamic range for all the images is 60 dB. The circular regions of interest (ROI) indicates where the contrast metrics were evaluated.

Finally, the array assembly was tested on a volunteer to image the liver (following the Stanford Institutional Review Board and after receiving informed written consent). The arrays were positioned under the ribcage to offer a transverse view of the liver. Two acquisitions collected at two different locations are given in Fig. 7, comparing the single array image with its three-array counterpart. The first noticeable difference was the enhanced field-of-view for the three-array image with multiple vascular landmarks detected. The improved lateral resolution gave a crisp image and the calculated contrast on regions of interest yielded significant improvements (Table 3). The SVD beamformer further enhanced the image quality, especially in the presence of a complex set of tissue layers above the liver as observed for the center section of the second acquisition (Fig. 7g–l). The close-up view in this case demonstrated poor contrast and the vessel boundaries were difficult to establish with the central array only (Fig. 7j) but were identifiable with three arrays (Fig. 7k,l).

**Table 3:** Evaluation of contrast ratio (CR), contrast-to-noise ratio (CNR) and generalized contrast-to-noise ratio (gCNR) in the liver from the acquisition shown in Fig. 7.

	1 array	3 arrays	3 arrays + SVD
**ROI 1**			
CR (dB)	− 6.0	− 8.7	− 11.2
CNR	0.79	1.07	1.25
gCNR	0.72	0.81	0.87
**ROI 2**			
CR (dB)	2.1	− 4.8	− 9.3
CNR	− 0.28	0.65	1.01
gCNR	0.68	0.72	0.80

## Discussion

Several groups have studied the use of multiple arrays for improving imaging through coherent beamforming but these configurations have not shown to be easily translatable to in vivo imaging where fast acquisition and practicality are required. A mechanical scan or angular sweep is the most straightforward approach^7,18^ but coherent beamforming is likely limited to static targets, reducing its usefulness in vivo. In the work from Peralta et al.^9^, two linear arrays (lambda pitch) are employed with a relatively large gaps between the arrays. The limited steering capabilities of linear arrays means that a relatively narrow beam overlapping area is available and the gaps can greatly affect the image quality if not reduced or corrected^8^. Moreover, transmitting with subapertures (i.e. only one array is active on transmit) increases data collection time. When incoherent compounding is realized from multiple views, contrast is improved as demonstrated for aorta imaging^13,14^ but resolution is unaffected. Here, the configuration of three phased arrays assembled together in a fixed geometry and interfaced to an ultrafast high-channel count ultrasound system provides an interesting platform to analyze in situ the potential benefits of large apertures. This setup considers the assembly of arrays as a single large aperture and provides real-time capabilities adapted to clinical imaging.

For this proof of concept study, the geometry cannot be changed in real time (as compared to^9,10^) but this choice simplifies use as the handling is similar to a commercial array. The 3D-printed manifold can be modified to provide other geometries (different curvature, more or fewer transducers, etc.) and tailored depending on the application. More arrays could increase the field of view and the stackable design was intended for this purpose. The ultrasound system in its current configuration allows control of up to 1024 elements (e.g. 8 arrays each with 128 elements) and extended configurations employing more probe will be tested in a follow-up study.

We chose to maintain the physical integrity of the array enclosure to maintain the factory electrical insulation for safe human imaging. Consequently, including a 15° angle between arrays was the simplest method to reduce the inter-array gaps. Aligning the arrays along a line would increase the gaps, widening the associated near-field dead zone and increasing the first grating lobe (Supplementary Fig. 2). Analysis of the piece-wise concave geometry also showed minimal difference compared to a flat array with the same lateral aperture (Supplementary Fig. 3). The overall dimensions of the assembly did not affect handling for transverse imaging of the liver but other orientations could be more challenging as the skin to probe contact could be harder to maintain. We envision that such a piece-wise concave array could perform well for fetal imaging where the geometry will match the natural skin curvature above the fetus.

It has been previously reported that for abdominal imaging (where ultrasound can travel through heterogeneous layers), a large aperture is desirable to improve lateral resolution and contrast, especially for deep targets^20^. We have demonstrated that the lateral resolution is greatly enhanced both in vitro on wire targets and on a commercial phantom, and more importantly in vivo on the liver. The improvement (× 3.4 at a depth of 100 mm) is consistent with the *f*-number reduction offered by the 99.4 mm aperture compared to the 27.9 mm aperture of a single P6-3 probe and results in a fine speckle texture. By implementing a PW sequence mimicking imaging with a single large aperture (such that all arrays are transmitting and receiving for each PW), we can also take advantage of ultrafast imaging by quickly scanning the enlarged field-of-view. This is an important aspect compared to conventional focused beam scanning as the larger field-of-view combined with the improved lateral resolving power would here require a large number of scan lines. The acquisition time would scale up, ultimately affecting the image quality in presence of tissue motion. In addition to the image improvement, the enlarged field-of-view is well suited for whole organ imaging and would be particularly useful for fetal and pediatric imaging.

Posterior acoustic shadowing^42^ or enhancement^43^ are clinically-useful features of suspicious lesions. The extended aperture imaging with plane waves, as applied here, is likely to reduce these artifacts. We cannot yet comment on how a reduction of these imaging artifacts could affect interpretation by a radiologist but in certain scenarios where posterior shadowing is detrimental to imaging, the extended aperture could help to image beyond the obstacle^44^. Further, if needed subaperatures can be used to assess the region of interest.

The axial resolution is unchanged which results in unusual PSFs with better lateral than axial resolution throughout most of the field-of-view as seen in the phantom data presented here. The P6-3 probe is an older design compared to state-of-the-art arrays providing better axial resolution (increased bandwidth) and better axial performance can be expected with a better performing probe. Similarly, the elevation resolution is unchanged which is an important aspect for the detection of small three-dimensional lesions^45^. The reduced improvements seen for the contrast on the liver are likely linked to the elevation resolution affecting the in-plane performance of the extended aperture. By comparison, elevation beam width is not affecting the results on the commercial phantom as all the targets are continuous in the elevation dimension. We can therefore envision even better performances with a 1.5D or 1.75D extended aperture allowing electronic focusing in elevation. Current developments in array architecture are paving the way to high performance large apertures^46^.

We have shown in vitro that in the absence of aberration on a calibrated phantom, the contrast of our extended aperture with a PW sequence is not better than using a single P6-3 array. It has been reported for simulated data that for a full synthetic aperture sequence, the contrast improves with the imaging aperture size^20^. The results of our study suggest that the improvement in lateral resolution comes at the cost of reduced coherence length and impacts contrast. For the PW sequence that was implemented, the angular range varies from − 30° to 30°. The result is consistent with the fact that plane wave transmits at widely separated angles have reduced coherence^47^. A detailed analysis of angular coherence in this large aperture configuration would fall outside of the scope of this study but should be informative regarding the trade-offs between lateral resolution and contrast.

Nevertheless, this PW approach provides a direct method for applying aberration compensation methods relying on ultrafast imaging. This process inherently optimizes coherence between the different PW transmits by removing low coherence signals. It is thus a helpful tool to compensate for the effect of the large aperture while maintaining the improved lateral resolution. When a real aberration is introduced, the piece-wise linear geometry that we tested shows a rapid degradation in image quality when the wavefront originates from different arrays (i.e. three angles is more degraded than a single PW transmit). The SVD beamformer breaks this dynamic and greatly improves contrast.

On wire targets and on the commercial phantom, the auto-regressive filter approach was effective at predicting data in the gaps and thus at reducing the associated grating lobes. However, estimating the filter coefficients and estimating the receive data for every receive event and for each pixel of the image significantly increases the processing time and currently prevents real-time operation. Dealing with sparse or partially sampled datasets is the subject of active research and machine learning approaches have proven to be good candidates to recover missing data^48,49^. We envision that such approach could be ideal in a multi-array configuration to recover missing data or compensate for gaps in real-time.

Finally, the maximum acquisition rate with the current system configuration is 250 us between successive plane wave transmits (corresponding to a pulse repetition frequency of 4000 Hz). Thus, for the 91-plane wave sequence presented in the manuscript, data acquisition is completed in 22.75 ms. Ultrafast applications (such as elastography^29^ or ultrafast ultrasound localization microscopy^36^) are not constrained by the extended aperture configuration. The implementation of partial GPU beamforming brought the reconstruction time to ~ 1 ms per plane wave (650 × 600 pixels, 130 × 120 mm^2^), and less than 100 ms per image with 91 plane waves. A sufficient video rate was achieved to easily manipulate the assembly in vivo and will be further optimized in the future.

## Methods

### Multi-array assembly

The manifold was designed in house based on 3D scans of the array enclosure. Each manifold is stackable to allow the addition of multiple arrays. A 15° angle was set between adjacent manifolds to minimize the inter-array gap. With three arrays assembled, the aperture extends over 99.4 mm with physical gaps of 9 mm and describes a segmented concaved array (Fig. 1a). In this work, we used a 128-element phased array (P6-3, ATL/Philips) with an inter-element pitch of 0.218 mm and an aperture size of 27.9 mm giving a total of 384 elements for the assembly. A phased array is suited for this configuration due to its wide acceptance angle (half-wavelength pitch) and more homogeneous performance compared to a linear array (wavelength pitch)^50,51^. Assuming the gaps were filled with physical elements by extending the segments formed by the individual P6-3, the assembly would contain 465 elements with an extra 40 and 41 elements in each gap respectively. The arrays were each connected to a Vantage 256 (Verasonics, Kirkland, WA), all belonging to the volumetric imaging package configuration consisting of four synchronized Vantage 256 with their respective host (Fig. 1b). This configuration allows to evenly split the load to 128 channels per system.

After assembly, the array positions were calibrated using the echoes from a 2-wire target immersed in water. To calibrate the location of the transducer elements, each P6-3 array transmitted a pulse to insonify two static wires (25 µm diameter spaced by ~ 15 mm) and recorded the backscattered echoes. The targets were immersed in water, placed at the center of the field-of-view and oriented to avoid overlap between the echo traces. The delay associated to each target was estimated with cross-correlation between the channels. The relative position (x, z) of the two targets with respect to the transmitting array was then recovered by fitting the delay trace associated with the target position (x, z) to the measured delays. The water speed-of-sound was set based on the measured temperature^52^ and correction for the acoustic lens was applied assuming a speed of sound of 1020 m/s and thickness of 1.38 mm. After determining the target location with respect to each of the three arrays, translation and rotation were applied to each array location to obtain their absolute position with respect to the central array.

We adjusted the elevation (out-of-plane) alignment of the arrays imaging plane through elevation hydrophone scanning. A needle hydrophone (HNP-0400, Onda, Sunnyvale, CA) was positioned on a linear stage at a distance of 130 mm. The elevation orientation of the outer arrays was adjusted to coincide with the central array imaging plane (Supplementary Fig. 4).

### Imaging sequence

The imaging sequence consisted of transmitting plane waves by forming a coherent wavefront with all three arrays (Fig. 1c) and receiving backscattered signals with the entire aperture (i.e. transmitting and receiving with all 384 elements). The transmitted plane waves are angled with respect to the central array which defines the global referential. The delays for the outside arrays were adjusted according to their location and orientation with respect to the global referential (Fig. 1d). For the data presented in this work, a total of 91 PWs were transmitted to scan the field-of view with an angle ranging from − 30° to 30° (with no apodization on transmit). We compared the results of the array assembly with images acquired only with the central array (i.e. only the central array was transmitting and receiving). For comparison of lateral resolution on wire targets, a full synthetic aperture acquisition was implemented. For acquisitions on phantom and in vivo on the liver, the same sequence of 91 PWs, − 30° to 30° was employed.

The transmit waveform was a 1-cycle pulse at 4.0 MHz and the pulse repetition frequency was set at 4000 Hz (i.e. a delay of 250 µs between successive transmits). Radio-frequency signals for all 384 elements were recorded at a sampling frequency of 15.625 MHz. Delay-and-sum beamforming was implemented on a graphical processing unit (GPU; Titan RTX, Nvidia, Santa Clara, CA) with CUDA codes designed in-house and allowing real-time processing and display. The system is configured to have one GPU per Vantage system which was here processing 128 channels (3 GPUs processing 384 channels in total). Reconstruction was done on a uniform half-wavelength grid considering the transmit beam information and with a *f*-number of 1 on receive. All processing was implemented on Matlab R2020a (Mathworks, Natick, MA). With a uniform pixel size of λ/2 (0.2 mm) and a beamforming grid of 650 × 600 (130 × 120 mm^2^), the image for one plane wave was reconstructed in 1 ms.

In vivo imaging was performed on the liver on a healthy volunteer (34 years old) following the protocol approved by the Stanford Institutional Review Board (Protocol #44593) and informed written consent was received from the volunteer. All methods were performed in accordance with the relevant guidelines and regulations. The sequence was calibrated to ensure that both the Mechanical Index (MI) and spatial peak time average intensity (I_spta_) were below the FDA recommendations. The array was placed under the ribcage to image the liver in a transverse plane and acquisition was performed during the scan (real-time beamforming). For comparison purposes, the sequence collected both the central array only data and the three-array data.

### Evaluation of imaging metrics

The lateral resolution was measured by evaluating the point spread function (PSF) on wire targets (25-µm diameter tungsten wire, Alfa Aesar, Haverhill, MA) in degassed water. The PSF was evaluated at a depth of 100 mm when using the central array only, the array assembly and the array assembly with gap compensation. Simulated datasets were also included in the analysis with the addition of a completely populated array with the same geometry but in which no gaps were present (465 elements in total compared to 384 elements for the physical array). To simplify the integration between the imaging sequence and the reconstruction routine, the Verasonics build-in simulator generated the simulated receive channel data.

To better visualize the improvement in lateral resolution, a target containing several wires with set spacings (lateral steps of 4, 3, 2 and 1 mm and axial steps of 5, 4, 3, 2 and 1 mm) was imaged. The wire target was also positioned at a depth 100 mm.

We evaluated the contrast, contrast-to-noise ratio (CNR) and generalized contrast-to-noise ratio (gCNR)^53^ on a tissue mimicking commercial phantom containing hypo- and hyperechoic regions (model 040GSE, CIRS, Norfolk, VA) with an attenuation of 0.5 dB/MHz/cm. Note that due to the piecewise concave shape of the aperture, we prepared an agar-based standoff to couple the array with the flat surface of the commercial phantom. The standoff had the same speed-of-sound as the phantom (1540 m/s) and was prepared as described in^54^ (SiC and Al_2_O_3_ powder were omitted). The metrics were calculated after drawing circular regions of interest (ROI) over the lesion and background as:

$$Contrast=20{log}_{10}\frac{{\mu }_{in}}{{\mu }_{out}}$$

$$CNR=\frac{{\mu }_{out}-{\mu }_{in}}{\sqrt{{\sigma }_{out}^{2}+{\sigma }_{in}^{2}}}$$

where µ and σ are the mean and standard deviation respectively in the ROI, with *in* indicating the lesion and *out* the background. The gCNR was calculated as:

$$gCNR = 1 - \int\limits_{{ - \infty }}^{{ + \infty }} {\mathop {{\text{min}}}\limits_{x} \left\{ {p_{{out}} \left( x \right),p_{{in}} \left( x \right)} \right\}dx}$$

where *p(x)* indicates the probability density function within the ROI.

### Gap compensation with the auto-regressive filter

Because of the physical gaps between the arrays, the large aperture is incomplete, resulting in grating lobes. We adapted here a method of gap compensation by creating virtual elements on receive for which the signals can be predicted with an autoregressive filter from the signals on the surrounding physical elements. The prediction filter is based on the auto-regressive model proposed by Shin et al.^41^. Briefly, it is assumed that the frequency-domain signal at the (*n* + 1)th channel, *S*_*f*_ (*n* + 1) can be expressed as a linear combination of the signals at the *p* preceding channels as:

$${S}_{f}\left(n+1\right)={{a}_{f}\left(1\right)S}_{f}\left(n\right)+{{a}_{f}\left(2\right)S}_{f}\left(n-1\right)+\cdots +{{a}_{f}\left(p\right)S}_{f}\left(n-p+1\right)$$

where *a*_*f*_ are the filter coefficients at each frequency *f*. After determining the coefficients *a*_*f*_ from the received signals, the physical arrays are extended by creating virtual elements in the gaps. We positioned the virtual elements by extending the segments defined by each array until intersection which simplifies the estimation process. With the array assembly described in this work, the gaps were filled with 81 virtual elements (24 extending array 1 on its right side, 16 and 13 extending array 2 on its left and right side respectively, and 28 extending array three on its left side).

The processing steps are summarized as follow. For each pixel, we apply delays on channel data *s* and select a time window of 4 wavelengths (16 time samples). We then perform the 1D FFT on *s*, noted *S*, and we estimate the coefficients *a*_*f*_ for each frequency in the transducer bandwidth with *p* = 8. The coefficient estimation was done with the built-in Matlab function *lpc* from the Signal Processing Toolbox. We can then add empty channels (virtual elements) to the channel data matrix and apply the AR filter at each frequency to estimate the signals in the gaps. This process is repeated for all the pixels in the image and is thus computationally intensive. It is not part of the real-time routine and is applied after the data is acquired.

The performance of the method was evaluated on the same wire target as described in the previous section. On simulated data, we also compared the gap compensation method with the equivalent fully populated aperture (i.e. an array of 465 elements with no gaps).

### Aberration correction with the SVD beamformer

The use of global plane waves with the array assembly has the benefit of allowing implementation of available aberration correction methods like the SVD beamformer^40^. Its implementation yields both an optimized image and a local estimation of the aberrations while filtering out low coherence signals.

Briefly, from an acquisition of *N* PWs, the method relies on optimizing coherence from the so-called Ultrafast Compound Matrix *R* containing *N* beamformed images (one for each angle) of *N*_*x*_ × *N*_*z*_ pixels. Isoplanatic patches are defined within the imaged field-of-view and SVD is performed on each patch. From the first singular vector, the rephased patch can directly be obtained. The final corrected image is computed after repeating the process by moving the patch over the entire image and assembling the corrected patches. A detailed analysis of the method can be found in^40^.

Aberration correction was first tested in silico on experimental data acquired on the calibrated phantom (model 040GSE, CIRS). A random numerical phase screen was applied on the radio-frequency dataset to degrade the quality of the delay-and-sum beamformed image. An experimental aberration was then introduced by replacing the trapezoidal agar/glycerol standoff (1540 m/s) with a water/ethanol solution (35/65 volume ratio) with a speed-of-sound of 1450 m/s^55^. The SVD beamformer was applied on the Ultrafast Compound Matrix for all datasets using a uniform patch size of 40λ × 40λ (15.4 × 15.4 mm^2^).

## Supplementary Information

Supplementary Figures.
