# Supplementary material for: Improving plane wave ultrasound imaging through real-time beamformation across multiple arrays
Source: Sci Rep. 2022 Aug 4;12:13386. doi: 10.1038/s41598-022-16961-2 (PMC9352764; doi:10.1038/s41598-022-16961-2)
Supplement: Supplementary file 1 — Supplementary Figures. [file 41598_2022_16961_MOESM1_ESM.docx]

Supplementary Information
Improving plane wave ultrasound imaging through real-time beamformation across multiple arrays

Josquin Foiret^1^, Xiran Cai^1^, Hanna Bendjador^1^, Eun-Yeong Park^1^, Aya Kamaya^1^, Katherine W. Ferrara^1^, ^1^Stanford University, Palo Alto, CA, USA.


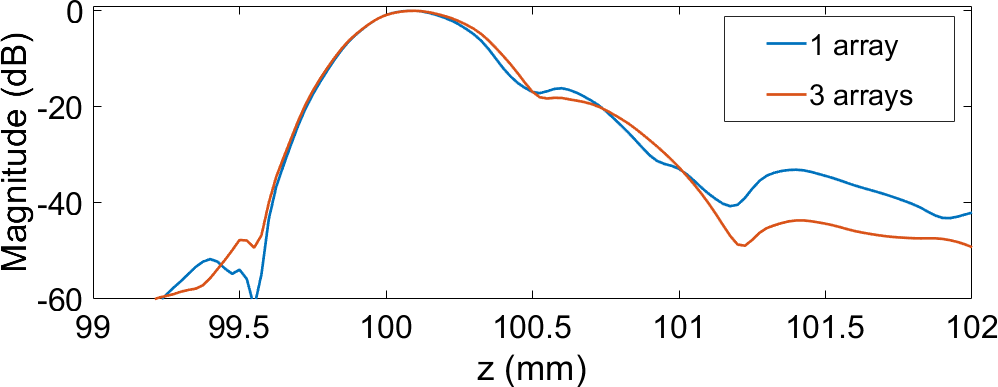


Supplementary Figure 1. Axial resolution is independent from the aperture size. The axial cross-section of the PSF at a depth of 100 mm (see Figure 1a) is similar imaging with one or three arrays.


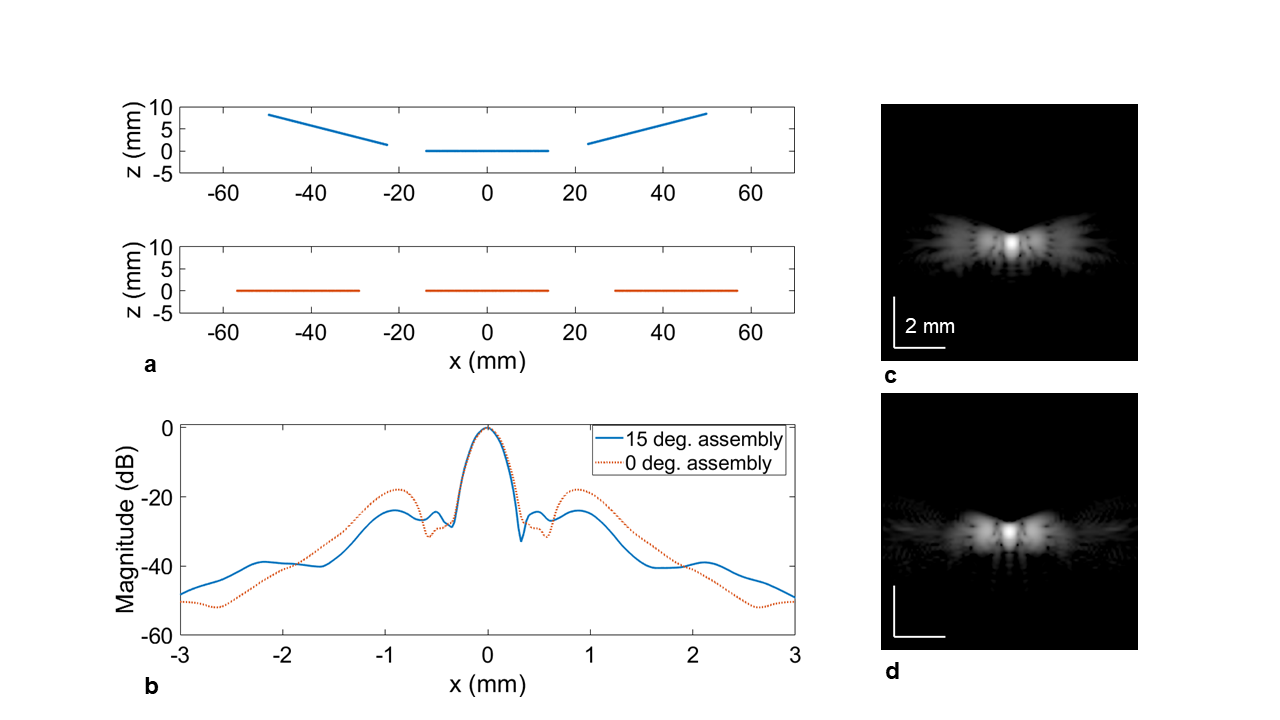


Supplementary Figure 2. Maintaining the integrity of each array enclosure requires trade-offs for the assembly imaging performance. (**a**) Minimum inter-array gaps are obtained with a 15° angle between arrays (top) with gaps of 9 mm. If the arrays are aligned on the same line (bottom), the inter-array gaps grow to 15.3 mm. (**b**) The lateral cross-section of simulated PSFs at a depth of 100 mm (51 PW, -30° to 30°) indicates a similar main lobe but a higher first grating lobe in the presence of larger inter-array gaps. (**c**)-(**d**). PSFs for the cross-section given in B ((**c**) 15° assembly, (**d**) 0° assembly). Images are displayed with a 60-dB dynamic range.


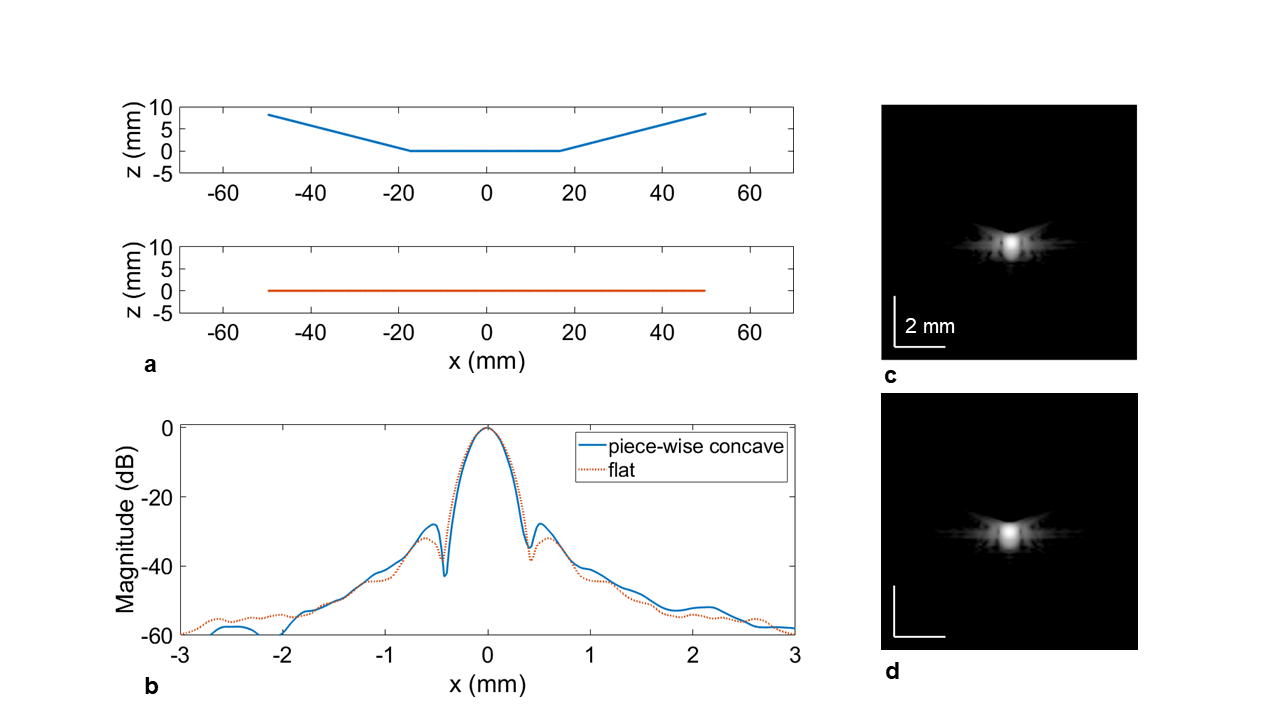


Supplementary Figure 3. For a given lateral aperture, a piece-wise concave or flat geometry yield very similar imaging performances. (**a**) A 15° piece-wise concave array (top) similar to the array utilized in the manuscript but with no gaps is compared to a flat array (bottom). Both arrays have the same lateral aperture of 98 mm. (**b**) The lateral cross-section of simulated PSFs at a depth of 100 mm (51 PW, -30° to 30°) indicates a similar main lobe and slightly degraded first grating lobe for the piece-wise concave array. (**c**)-(**d**) PSFs for the cross-section given in B ((**c**) piece-wise concave, (**d**) continuous array). Images are displayed with a 60-dB dynamic range.


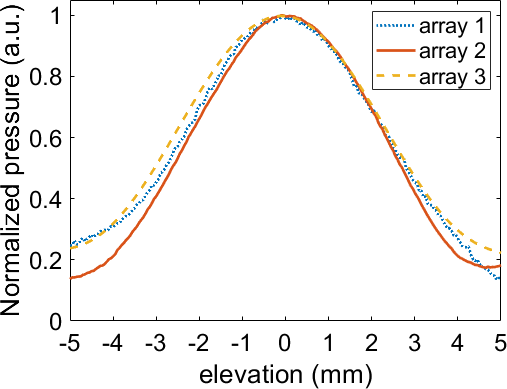


Supplementary Figure 4. The out-of-plane alignment of the three arrays was checked with hydrophone scans in the elevation plane. The normalized pressure plots for elevation scans realized at a depth of 130 mm indicates a coincidence of the imaging planes (maximum pressure).
